# Artificial intelligence as a door opener for a new era of human reproduction

**DOI:** 10.1093/hropen/hoad043

**Published:** 2023-11-25

**Authors:** Markus Hengstschläger

**Affiliations:** Institute of Medical Genetics, Medical University of Vienna, Vienna, Austria

Sir,

The first successful IVF birth in 1978 laid the foundation for the selection of human embryos prior to their transfer into a uterus. The optimization of this selection process is viewed as crucial to counter the low success rates of IVF, which is considerably affected by the failure of the majority of the transferred embryos to implant. What originally started with non-invasive morphological quality assessment and morphokinetic analysis of embryos, progressed to aneuploidy screening and testing for single gene disorders on biopsied embryonic cells and has already reached the level of screening for polygenic conditions and traits (Turley *et al.*, 2021). While related pros and cons are still under discussion, the idea of a combined genomic, transcriptomic, proteomic, and metabolomic embryo assessment has already come to the fore. To our best knowledge, the excellent article by Salih *et al.* (2023) is the first systematic demonstration that artificial intelligence has the potential to outperform non-invasive embryo selection via visual analysis by embryologists. Here, I would like to express my conviction that the convergence of recent groundbreaking scientific developments could provide the tools to surmount the main obstacles for the simultaneous use of non-invasive multi-parameter embryo assessments and invasive multi-omics applications to enhance the low global success rates of IVF.

First, the approaches mentioned above can only reveal their true benefits if patients have a high number of embryos to choose from. In the future, the low number of natural oocytes matured upon hormone stimulation might be superseded by limitless quantities of gametes developed *in vitro* from induced pluripotent stem cells generated from parental skin cells. Even functional stem cell-derived oocytes from male mice have already produced fertile offspring (Murakami *et al.*, 2023). To date, although only rudimentary human gametes have been able to be generated and several safety and ethical issues still need to be resolved, biomedical companies, well-funded by venture capital, have already predicted the translation of *in vitro* derived oocytes and sperm to human reproductive medicine within the next few years (Cyranoski *et al.*, 2023).

Second, as also highlighted by Salih *et al.* (2023), artificial intelligence is on its way towards entering routine clinical use. Although large trials to evaluate efficacy are yet to be carried out, non-invasive deep-learning approaches using time-lapse microscopy pictures in combination with different clinical parameters such as maternal age have very recently been shown to be useful for the selection of the most viable embryos for transfer (Barnes *et al.*, 2023). In addition, in molecular medicine, artificial intelligence has already paved the path for the interpretation of data obtained upon multi-omics applications and certainly enables the amalgamation of this with other types of multidimensional medical data (Gomes and Ashley, 2023).

Without doubt, the convergence of these technologies will herald a new era of embryo selection in reproductive medicine with putative consequences for our idea of human origins. For the benefit of both patients and clinicians, its foreseeable clinical translation must be accompanied by an intensive ethical deliberation (Horer *et al.*, 2023).
